# Advancing an interdisciplinary framework to study seed dispersal ecology

**DOI:** 10.1093/aobpla/plz048

**Published:** 2019-08-19

**Authors:** Noelle G Beckman, Clare E Aslan, Haldre S Rogers, Oleg Kogan, Judith L Bronstein, James M Bullock, Florian Hartig, Janneke HilleRisLambers, Ying Zhou, Damaris Zurell, Jedediah F Brodie, Emilio M Bruna, Robert Stephen Cantrell, Robin R Decker, Edu Efiom, Evan C Fricke, Katherine Gurski, Alan Hastings, Jeremy S Johnson, Bette A Loiselle, Maria N Miriti, Michael G Neubert, Liba Pejchar, John R Poulsen, Gesine Pufal, Onja H Razafindratsima, Manette E Sandor, Katriona Shea, Sebastian Schreiber, Eugene W Schupp, Rebecca S Snell, Christopher Strickland, Jenny Zambrano

**Affiliations:** 1 Department of Biology & Ecology Center, Utah State University, Logan, UT, USA; 2 Landscape Conservation Initiative, Northern Arizona University, Flagstaff, AZ, USA; 3 Department of Ecology, Evolution, and Organismal Biology, Iowa State University, Ames, IA, USA; 4 Physics Department, California Polytechnic State University, San Luis Obispo, CA, USA; 5 Department of Ecology and Evolutionary Biology, University of Arizona, Tucson, AZ, USA; 6 Centre for Ecology and Hydrology, Benson Lane, Wallingford, UK; 7 Theoretical Ecology, University of Regensburg, Regensburg, Germany; 8 Biology Department, University of Washington, Seattle, WA, USA; 9 Department of Mathematics, Lafayette College, Easton, PA, USA; 10 Swiss Federal Research Institute WSL, Dept. Land Change Science, Birmensdorf, Switzerland; 11 Humboldt-University Berlin, Geography Dept., Berlin, Germany; 12 Division of Biological Sciences and Wildlife Biology Program, University of Montana, Missoula, MT, USA; 13 Department of Wildlife Ecology & Conservation & Center for Latin American Studies, University of Florida, Gainesville, FL, USA; 14 Department of Mathematics, The University of Miami, Coral Gables, FL, USA; 15 Department of Environmental Science and Policy, University of California, Davis, CA, USA; 16 REDD+ Unit, Cross River State Forestry Commission, Calabar, Nigeria; 17 Biology Department, Lund University, Lund, Sweden; 18 National Socio-Environmental Synthesis Center, University of Maryland, Annapolis, MD, USA; 19 Department of Mathematics, Howard University, Washington, DC, USA; 20 Santa Fe Institute, Santa Fe, NM, USA; 21 School of Forestry, Northern Arizona University, Flagstaff, AZ, USA; 22 Center for Latin American Studies and Department of Wildlife Ecology and Conservation, University of Florida, Gainesville, FL, USA; 23 Department of Evolution, Ecology and Organismal Biology, The Ohio State University, Columbus, OH, USA; 24 Department of Biology, Woods Hole Oceanographic Institution, Woods Hole, MA, USA; 25 Department of Fish, Wildlife and Conservation Biology, Colorado State University, Fort Collins, CO, USA; 26 Nicholas School of the Environment, Duke University, Durham, NC, USA; 27 Natur Conservation and Landscape Ecology, University of Freiburg Freiburg, Germany; 28 Department of Biology, College of Charleston, Charleston, SC, USA; 29 Department of Biology, The Pennsylvania State University, University Park, PA, USA; 30 Department of Evolution and Ecology and Center for Population Biology, University of California, Davis, CA, USA; 31 Department of Wildland Resources & Ecology Center, Utah State University, Logan, UT, USA; 32 Department of Environmental and Plant Biology, Ohio University, Athens, OH, USA; 33 Department of Mathematics, University of Tennessee Knoxville, Knoxville, TN, USA; 34 Department of Biology, University of Maryland, College Park, MD, USA

**Keywords:** Analytical models, demography, global change, individual-based models, long-distance seed dispersal, population models, seed dispersal

## Abstract

Although dispersal is generally viewed as a crucial determinant for the fitness of any organism, our understanding of its role in the persistence and spread of plant populations remains incomplete. Generalizing and predicting dispersal processes are challenging due to context dependence of seed dispersal, environmental heterogeneity and interdependent processes occurring over multiple spatial and temporal scales. Current population models often use simple phenomenological descriptions of dispersal processes, limiting their ability to examine the role of population persistence and spread, especially under global change. To move seed dispersal ecology forward, we need to evaluate the impact of any single seed dispersal event within the full spatial and temporal context of a plant’s life history and environmental variability that ultimately influences a population’s ability to persist and spread. In this perspective, we provide guidance on integrating empirical and theoretical approaches that account for the context dependency of seed dispersal to improve our ability to generalize and predict the consequences of dispersal, and its anthropogenic alteration, across systems. We synthesize suitable theoretical frameworks for this work and discuss concepts, approaches and available data from diverse subdisciplines to help operationalize concepts, highlight recent breakthroughs across research areas and discuss ongoing challenges and open questions. We address knowledge gaps in the *movement ecology* of seeds and the integration of *dispersal and demography* that could benefit from such a synthesis. With an interdisciplinary perspective, we will be able to better understand how global change will impact seed dispersal processes, and potential cascading effects on plant population persistence, spread and biodiversity.

## Teaser

Although dispersal is generally viewed as a crucial determinant for the fitness of any organism, our understanding of its role in the persistence and spread of plant populations remains incomplete. In this perspective, we provide guidance on integrating empirical and theoretical approaches that account for the context dependency of seed dispersal to improve our ability to generalize and predict the consequences of dispersal, and its anthropogenic alteration, across systems. With an interdisciplinary perspective, we will be able to better understand how global change will impact seed dispersal processes, and potential cascading effects on plant population persistence, spread and biodiversity.

## Introduction

Dispersal influences individual fitness (Saastamoinen *et al.* 2018), population persistence (Kendrick *et al.* 2012) and biodiversity across scales (Vellend 2010), as well as a population’s ability to track shifting habitats, deal with large-scale environmental variability and adapt to novel environments in response to global change (Zhou and Kot 2011; Clobert *et al.* 2012; Travis *et al.* 2013). Global change, including climate change, habitat fragmentation and overharvesting, affects the ecology and evolution of dispersal, in turn altering the ability of species to move or adapt to global change events (Travis *et al.* 2013). For sessile organisms such as plants, dispersal of propagules—defined as the movement away from the parent location—may be the sole opportunity to escape changes in local environmental conditions. Ecological understanding of dispersal has progressed by describing patterns of dispersal and the conditions under which they arise (Nathan and Muller-Landau 2000; Nathan *et al.* 2012), advancing dispersal theory for populations and communities (Levin *et al.* 2003; Levine and Murrell 2003) and determining the effectiveness of seed dispersal (Schupp 1993; Schupp *et al.* 2010, 2017). Nevertheless, the role of seed dispersal in the long-term spatial dynamics of plant populations remains poorly understood. The complexity and context dependence of seed dispersal ecology challenges our ability to generalize across different systems and predict responses of plant diversity to global change. To move towards the predictive understanding necessary to inform conservation strategies requires a systematic examination of dispersal mechanisms and their influence on the persistence and spread of populations.

Seed dispersal ecology is complex and context-dependent (Schupp 2007; see Figure 1 in Beckman *et al.* 2020). Plants exhibit a diverse array of strategies to disperse their propagules using biotic and abiotic vectors. The majority of plants are dispersed by animals (56 %; Aslan *et al.* 2013), including mammals, birds, reptiles and ants; some self-disperse, such as through ballistic action, and the rest are dispersed by abiotic means, including wind, water and gravity. Dispersal vectors affect seed viability and the temporal and spatial patterns of seed rain, which influences the ‘seedscape’, i.e. the abiotic and biotic environments surrounding a seed that influence later recruitment stages (Beckman and Rogers 2013). The pattern of seed deposition determines a plant’s interactions with neighbours competing for limiting resources, the likelihood of mortality due to natural enemies, the possibility of avoiding catastrophic losses due to disasters and the potential of reaching microsites suitable for survival, growth and future reproduction (Howe and Smallwood 1982; Schupp and Fuentes 1995; Nathan and Muller-Landau 2000; Beckman and Rogers 2013). For most plants, mortality is highest during the early stages of the life cycle, and the vast majority of seeds do not lead to a reproductive adult (Terborgh *et al.* 2014). Ecological processes from seed production to recruitment thus determine gene flow and the colonization of new areas, ultimately influencing the spatial distribution of species, community diversity and ecosystem functioning.

Our incomplete understanding of seed dispersal’s role in plant populations stems from seed dispersal ecology being largely based on short-term, local-scale empirical studies for a small number of species, on the one hand, and, on the other hand, theoretical dispersal models that often make simplified assumptions, bringing into question their suitability for making quantitative and system-specific predictions. These barriers exist for several reasons. First, seed dispersal is only one process in the chain of events within a plant’s life cycle (from flower to reproductive adult), and it interacts with several other processes over multiple spatiotemporal scales. Consequently, it is difficult to quantify the demographic importance of dispersal relative to processes affecting survival and growth at later life-history stages. Second, options for controlled experiments are limited because of the difficulty of manipulating dispersal at the spatial, temporal or organizational scales relevant to assess its complete demographic impact (e.g. Augspurger and Kitajima 1992; Coulson *et al.* 2001; Poulsen *et al.* 2012). Third, uncovering spatial processes from available observational data on spatial patterns of plant recruitment necessitates the collection of detailed field data to isolate different processes that result in similar patterns (e.g. Wiegand *et al.* 2009). Fourth, analysing mathematical or simulation models based on realistic assumptions of processes occurring across multiple spatiotemporal and organizational scales and in heterogeneous environments requires mathematical and statistical rigor within an interdisciplinary context (e.g. Harsch *et al.* 2014). To overcome these challenges and improve our ability to understand and predict the contributions of seed dispersal to populations requires a comprehensive framework that quantitatively integrates dispersal and demography. In other words, we need to evaluate the impact of any single seed dispersal event within the full spatial, temporal and environmental context of a plant’s life history to fully understand the contribution of seed dispersal to population dynamics, thereby closing the seed dispersal loop (Wang and Smith 2002).

**Figure 1.**
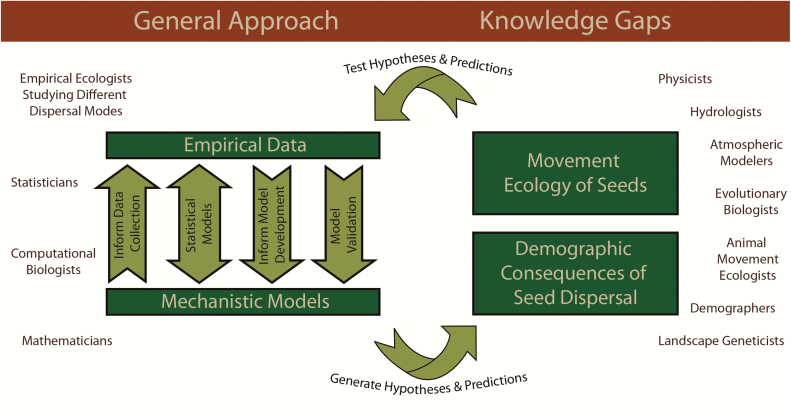
To advance current knowledge gaps in seed dispersal ecology requires interdisciplinary collaboration in which researchers simultaneously and iteratively collect empirical data and develop mechanistic models that are integrated with statistics.

**Figure 2.**
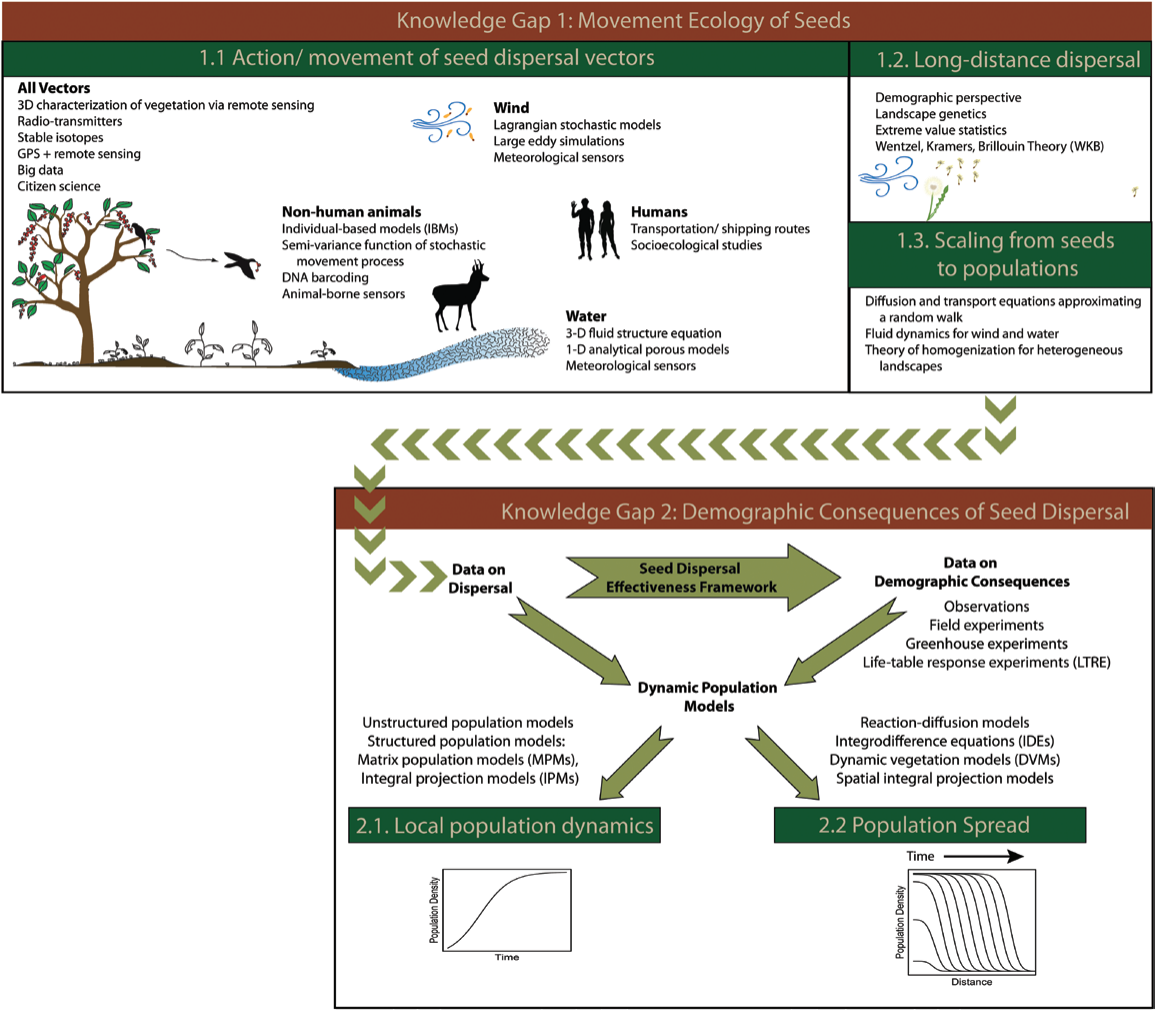
Examples of the differing empirical and modelling approaches used to quantify dispersal and estimate the impacts of dispersal. We suggest that studies combining multiple approaches are likely to provide greater insight into dispersal dynamics.

Here, we discuss how the above goal can be reached (Fig. 1). We begin by providing a general perspective on integrating empirical and theoretical methods for addressing the context dependency of seed dispersal to generalize and predict across systems. We then highlight two knowledge gaps that could benefit from such an integrative approach. First, we present advances and challenges in the *movement ecology of seeds*, considering the multitude of seed dispersal mechanisms and vectors that influence spatial patterns of seed dispersal. Second, we discuss potential pathways for integrating *dispersal and demography* to reach an improved understanding of population persistence and population spread. Throughout, we demonstrate that advancing the study of seed dispersal and its influence on population dynamics requires increased collaboration among researchers that examine disparate life-history stages of plants from a variety of disciplinary, geographic and organismal perspectives. Such studies will be even more powerful if they take advantage of advances in empirical, statistical, computational and mathematical methods, in tandem with global initiatives and standardized experiments over large geographic extents. We propose promising multidisciplinary and interdisciplinary advances, including opportunities to apply existing frameworks and approaches from other disciplines to advance seed dispersal ecology (Fig. 2). We synthesize suitable theoretical frameworks for this work and discuss concepts, approaches and available data from diverse subdisciplines to help operationalize concepts, highlight recent breakthroughs across research areas and discuss ongoing challenges and open questions. We end with specific strategies to guide future research.

## A general approach for studying context dependence of seed dispersal

The large number of processes and agents that constitute dispersal (see Figure 1 in Beckman *et al.* 2020) create a distinct paradox: to predict the consequences of dispersal, we need to simultaneously reduce complexity to generalize across systems and embrace complexity to be able to make system-specific predictions (see also Evans *et al.* 2013). Reducing complexity can (i) aid in scaling across ecological organizational levels, (ii) reduce the need for data that may not be logistically feasible to collect and (iii) increase the efficiency of the computational models necessary for answering pressing conservation and management issues. By contrast, embracing complexity can (i) provide quantitative predictions for specific conservation and management issues and (ii) allow for a more faithful representation of a particular ecological system. The approach a researcher uses depends on their aim, that is, generalization across systems or specific forecasts, the question of interest and knowledge about the system, including available data.

### How to reduce complexity?

To move towards a more fundamental understanding of seed dispersal, we need to know when and how we can generalize dispersal and its impacts on populations. This requires both advances in the theory of dispersal ecology and standardized empirical methods to test and inform theory. Theory can take the form of conceptual, statistical, simulation or mathematical models and allows us to clearly formulate our assumptions and the expected first principles underlying observed patterns while necessarily simplifying the system of interest (Marquet *et al.* 2014). Theory and standardized data collection will aid us in finding differences and commonalities across systems and can help determine if, when, and how we can scale from local empirical studies to predict qualitative or quantitative responses to global change at larger temporal, spatial or organizational scales. Building and collaborating with international networks of researchers (Frugivory and Seed Dispersal: e.g. Estrada and Fleming 1986; Levey *et al.* 2002; Hardesty 2007, CoDisperse: e.g. Beckman *et al*. 2020) we can integrate theory with data from existing studies, long-term data sets and future data collection initiatives developed by an interdisciplinary network of researchers to answer the most pressing questions in seed dispersal ecology.

### How to embrace complexity?

To enhance system-specific predictions, we need to address uncertainty, boost simulation capacity and collect relevant ecological and natural history data. Systems-based approaches can be used to understand a system as a whole and to incorporate the complexity of ecosystems as well as uncertainty related to data, model structure and model selection (e.g. Hartig *et al.* 2012; Milner-Gulland and Shea 2017). We can include mechanistic representations of reproduction, dispersal, growth and survival that allow predictions of dynamic responses to future global change and novel conditions, without assuming static relationships under current environmental conditions. Connecting these models to data requires statistical advances, such as Bayesian Inference or Approximate Bayesian Computation (ABC; Hartig *et al.* 2011, 2012), that incorporate heterogeneous data into process-based models to reduce uncertainty and test model output with data. Additionally, development of systems-based approaches to study seed dispersal requires computational advances to deal with multi-scale problems, mathematical advances that can approximate complexity and reduce computational expenses, and integration of empirical data across systems and subdisciplines that study the movement of seeds, their corresponding vectors (e.g. wind, water, animals, etc.) and the fitness contributions of seed dispersal.

### Confronting complexity with models and data

We believe that a promising approach to confront the complexity and context dependency of seed dispersal is to allow for feedbacks between empirical observations and the exploration of dynamics by simultaneously and iteratively collecting data and developing models. This approach would allow for data to inform the development and refinement of model assumptions, parameters and structure, and for models to elucidate mechanisms driving empirical patterns. By collecting data on dispersal processes simultaneously with model development, we can use models to develop hypotheses and predictions that can be tested empirically, and with an iterative approach, we can refine models based on empirical results to develop and test new hypotheses and predictions. Proper incorporation of stochasticity can help determine the limits to prediction as well as experimental challenges. In addition, models can be developed based on the results from manipulative experiments and project the consequences of dispersal for higher organizational levels (e.g. populations, communities) or over a larger spatial and temporal extent than is possible with manipulative experiments alone. Results of these models can be compared to observational data to help discern whether and how dispersal processes lead to empirical patterns observed over larger spatiotemporal scales. Finally, mechanistic models can predict responses to different scenarios of novel conditions anticipated from global change models. Mechanistic models range from analytical models to complicated simulation models (Box 1; Dieckmann *et al.* 2000; Jongejans *et al.* 2008). In addition, phenomenological models can be useful in describing dispersal patterns (e.g. Bullock *et al.* 2017) and approximating mechanistic models of dispersal for inclusion in process-based models. Data collection efforts can include synthesis of existing knowledge or collection of data from manipulative experiments and observations from the field, greenhouse and laboratory. The most appropriate modeling approach depends on research questions, assumptions and type of data available (Box 1).

Box 1. **Overview of models**

**Figure 3.**
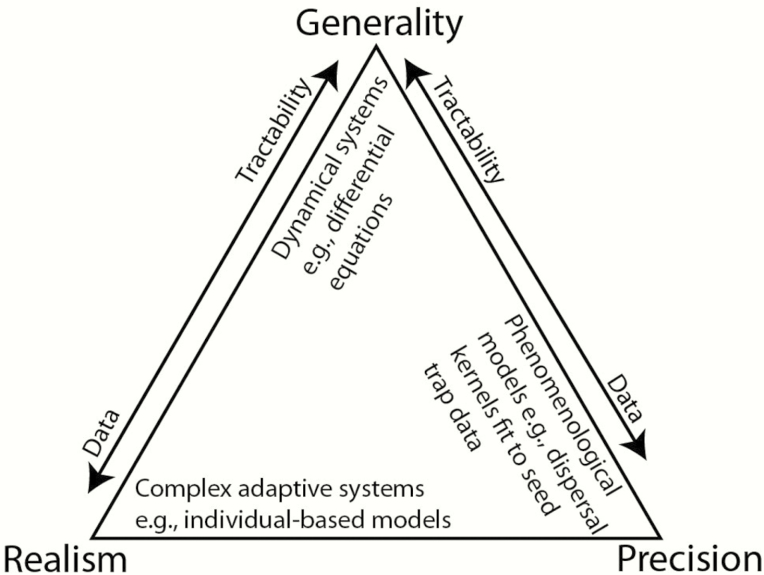
Trade-offs in model building as discussed by Levins (1966): the goals of models are to maximize generality, realism and precision but trade-offs exist such that only two of these three goals can be captured. While there is philosophical doubt on whether these trade-offs exist (Evans 2012), maximizing all three goals will likely result in a model that is intractable and impossible to analyse (Silverman 2018).

Developing and evaluating process-based models requires empirical studies to identify the processes to be included (model structure), the descriptions used for those processes (model selection) and data on parameters (Grimm and Railsback 2011). Based on the purpose of the model, researchers will need to decide how to balance generality, realism and precision (Fig. 3).

Analytical mathematical models offer conceptual insights on the qualitative behaviour of the system by using simplifying assumptions that allow the general contribution of different processes and parameters to be evaluated. This can be particularly helpful when data are limited (Bullock *et al.* 2012). Analytical models can also facilitate scaling from individual seeds to populations by approximating computationally expensive simulations while retaining key dispersal mechanisms (e.g. Travis *et al.* 2011). More complicated models that are fine-tuned for a specific system are thought to have greater predictive power (Evans *et al.* 2013), though this requires further investigation as adding more complicated model structure increases uncertainty (Sun *et al.* 2016). Simulation models, such as individual-based models (also known as agent-based models), are becoming more sophisticated as computing power increases and can be quite useful for suggesting how individual-level processes give rise to complex population-level phenomena. However, complicated simulations trade analytical tractability, computational inexpensiveness and fewer data requirements for direct incorporation of natural complexity, real-world variability and uncertainty (Fig. 3).

Further assumptions to consider during model development are whether, and how, to incorporate time, space, stochasticity and individuals. Does the question of interest involve static relationships or changes over time (i.e. static vs. dynamic models)? If researchers are interested in changes over time, do the entities in the model experience time continuously (e.g. overlapping generations) or discretely (e.g. seasonality), and what temporal scales are relevant? Is space important; should it be continuous or discrete, and what spatial scales are relevant? How important is it to consider deterministic vs. stochastic model versions? Can the system be modelled assuming large population sizes or are interactions among discrete individuals important to consider? Other questions to consider involve the detail of processes to be included. For example, does dispersal need to be represented by detailed movement pathways or are phenomenological dispersal patterns sufficient? What is the importance of demographic variation? How important are interactions with mutualists (e.g. mycorrhizae) and antagonists (e.g. competitors, natural enemies) at the site of deposition?

In summary, seed dispersal is a complex and context-dependent process, but we assert that the seed dispersal loop can be closed and the contribution of seed dispersal to plant population dynamics can be quantified from multidisciplinary and interdisciplinary perspectives. We can achieve this by synthesizing recent advances in analytical mathematical models, computational simulation models, statistics, data synthesis and coordinated data collection on dispersal and recruitment processes. Such an integration will ultimately help balance necessary complexity with tractability.

Next, we discuss advances and challenges in confronting this context dependency with data and models in the context of two knowledge gaps: (i) mechanisms underlying the movement ecology of seeds and resulting dispersal patterns and (ii) demographic consequences of this movement.

## Gap 1: Understanding the movement ecology of seeds

This first knowledge gap focuses on improving our mechanistic understanding of the movement of individual seeds in order to generalize dispersal mechanisms and patterns across systems and to predict dispersal under novel conditions. Studies uncovering spatial patterns of seed dispersal have tended to focus on population-level patterns (e.g. Eulerian methods), but are becoming increasingly mechanistic by focusing on the movement of individual seeds (e.g. Langrarian approaches; Turchin 1998). To describe population-level spatial patterns of seed dispersal, ecologists have estimated dispersal kernels (probability density function of dispersal distances; Nathan *et al.* 2012) by combining seed traps with inverse modeling (Nathan and Muller-Landau 2000) and incorporating genetic information from seeds and parents (e.g. Hardesty 2007; Jones and Muller-Landau 2008). While these analyses increase our understanding of the variation in seed dispersal patterns, dispersal kernels tend to be phenomenological (Nathan *et al.* 2012; but see Katul *et al.* 2005; Codling *et al.* 2008 for examples of mechanistically derived dispersal kernels) and therefore have limited capability for forecasting changes in dispersal itself under novel conditions resulting from global change. In addition, these phenomenological models tend to describe the spatial patterns of seeds arising from the final outcome of dispersal and not the process of dispersal, while a mechanistic understanding can only be achieved by partitioning the contributions of different dispersal vectors (see Rogers *et al.* 2019 for solutions to this issue).

A more mechanistic understanding of the movement of individual seeds requires explicitly quantifying the action and movement of different seed dispersal vectors and their interactions with plants. A challenge will be measuring the extent of long-distance dispersal (LDD), rare events that are particularly difficult to study but likely critical to the establishment of new populations, colonization after disturbance and rapid plant migration in response to climate change (Nathan 2006). Finally, we need to be able to scale up movements of individual seeds to effectively generalize and predict spatial patterns that emerge at the population level. Development of models informed by empirical data will help us incorporate the necessary level of complexity for dispersal vectors and their interactions with plants, measure the extent of LDD and scale from the movement of individual seeds to describe population-level spatial patterns.

### Action/movement of seed dispersal vectors

**Figure 4.**
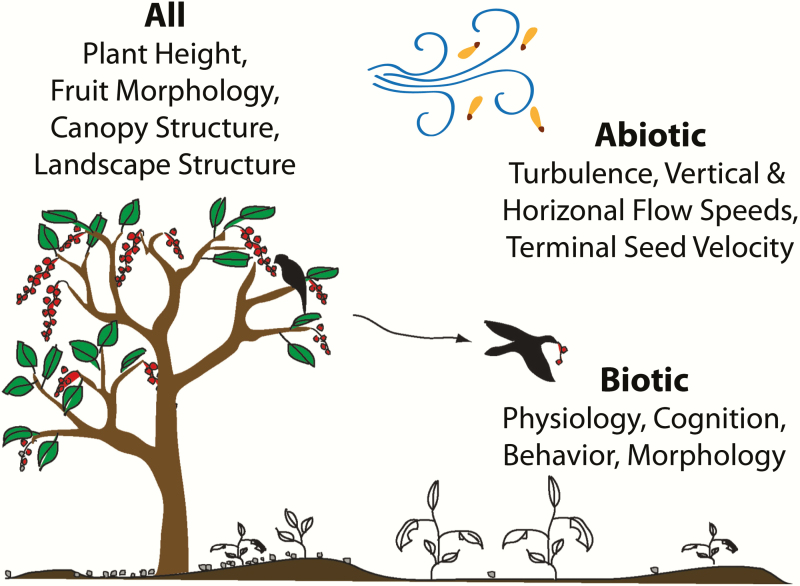
Examples of processes influencing abiotically and biotically dispersed seeds.

Across species, plants are dispersed by a range of dispersal vectors; even an individual seed may be dispersed by a suite of abiotic and biotic means. These vectors have different consequences for seed dispersal patterns and require a range of empirical and mathematical methods to uncover and describe associated processes (Fig. 4). Investigating all the actions, movements and processes influencing the journey of a seed at the plant, population or species level is daunting but a mechanistic understanding is possible by integrating empirical and theoretical approaches. One approach is to describe functional groups to generalize across species as discussed by Aslan *et al.* (2019). Another is to draw general lessons from analysis of total dispersal kernels for key species (Rogers *et al.* 2019). Here, we highlight data-driven quantitative approaches that enable researchers to describe these complex processes and advance a mechanistic understanding of different vectors, focusing on wind, water and animals, including humans.

For abiotically dispersed plant species, we can gain an increased mechanistic understanding of dispersal processes from physics and hydrology. The physics of the transport of propagules—such as spores, pollen and seeds—due to the action of wind or water is a vast field in its own right (e.g. Okubo and Levin 1989; Isard and Gage 2001; Katul *et al.* 2005; Nathan *et al.* 2011b; Aylor 2017). For wind dispersal, a typical seed dispersal event first involves release of a seed from the plant canopy. For plants in the herbaceous layer, seeds are generally dispersed above the canopy, and any seeds released under the canopy settle immediately due to very low wind speeds (Soons *et al.* 2004). For trees and shrubs, the seed will experience dispersal within the canopy due to canopy-scale turbulence, sometimes followed by escape from the canopy and transport via the surface layer or even higher levels of the atmospheric boundary layer, before being deposited (Augspurger 1986). Each of these steps involves turbulence and advective flows with different properties. Hence, one of the challenges is to connect dispersal processes that dominate at different scales (Pauchard and Shea 2006). A variety of numerical simulation methods have been developed, including Lagrangian stochastic models (Katul *et al.* 2005; Kuparinen 2006; Aylor 2017) and large eddy simulations (Chamecki *et al.* 2009; Nathan *et al.* 2011b). These mechanistic models, varying in levels of complexity, have given us insights on the importance of seed abscission, canopy structure, plant height and land surface heterogeneity on LDD through effects on turbulence and wind speed, but additional advances in theory are required to generalize across systems (Nathan *et al.* 2011b). For seed dispersal by water, obtaining a fine-scale resolution of flow requires numerically solving 3-D fluid-structure interaction equations, which is extremely expensive computationally. In other scenarios, 1-D analytical porous models may suffice to resolve flow through vegetative beds including sea grasses, reefs and macrophytes (e.g. Brinkman 1949; Strickland *et al.* 2017). These modeling approaches for abiotically dispersed plant species can be further developed with advances in data collection. For example, remote sensing now enables 3-D characterizations of vegetation (e.g. Lefsky *et al.* 2002; Eitel *et al.* 2016), and meteorological sensors (FLUXNET; Baldocchi *et al.* 2001) allow monitoring speeds and turbulence of wind and water at high spatial and temporal resolutions.

The dispersal of a seed by an animal depends on the vector’s life-history strategy, local abundances and distributions of dispersers and fruiting trees, landscape structure, and individual characteristics of the animal and fruit themselves (Nathan *et al.* 2008; Cortes and Uriarte 2012; Schupp *et al.* 2019; Snell *et al.* 2019). Spatially explicit individual-based models (IBMs) can integrate data on dispersal processes, such as gut retention time, animal movement and number of seeds dispersed, to determine the spatial locations of seeds and contribution to long-distance seed dispersal. For example, Kleyheeg *et al.* (2017) predicted seed dispersal patterns by the mallard (*Anas platyrhynchos*), an important dispersal vector of wetland plants, by using a spatially explicit, mechanistic simulation model developed from high-resolution data on gut-passage times and landscape-scale movements of the mallard. Pires *et al.* (2018) estimated that LDD reduced by at least two-thirds following extinctions of mammals in specific Pleistocene assemblages using a mechanistic simulation model incorporating seed ingestion, gut retention, animal movement and seed deposition. Animal movement relevant to seed dispersal can occur across multiple spatiotemporal scales; for example, an animal may forage at fine spatial and temporal scales but search for foraging sites at long distances. The movement path of an individual arises from an animal’s internal state, navigation capacity, motion capacity and the environment (Nathan *et al.* 2008). Multiple behaviours of animal movement are quantified using observational data on the locations of individual animals collected at predetermined fixed time intervals, and some of the derived quantities used to describe movement are sensitive to the choice of sampling rate. These sampling rates should be guided by the research question and the movement process under investigation. Fleming *et al.* (2014) recently developed an approach using a semi-variance function of a stochastic movement process that enables identification of multiple modes of animal movement that vary across spatiotemporal timescales (e.g. foraging, simple random search and home range) and provides a solution to the sampling rate problem. Using this approach, they were able to incorporate foraging behaviour into existing animal movement models. Finally, understanding the preference and avoidance of certain habitats within the landscape by animal seed dispersers will be necessary for determining subsequent growth and survival of plants after deposition. For example, Kleyheeg *et al.* (2017) found that landscape configuration governs mallard movements, and transport of seeds to core areas may help maintain connectivity of wetland plant populations.

To better understand the mechanisms of seed dispersal in socioecological systems, we need to consider both accidental and deliberate seed dispersal by humans (e.g. Wichmann *et al.* 2009; Taylor *et al.* 2012), which can occur over great distances that are potentially global in scale (Bullock *et al.* 2018). Methods are being developed to quantify and model seed dispersal by humans. Relevant advances in invasion biology include genetic analysis to identify seed sources (e.g. Eriksen *et al.* 2014), transportation/shipping route mapping (e.g. Miller and Ruiz 2014; Chapman *et al.* 2017) and socioecological studies of human behaviours and movements (e.g. Wilson *et al.* 2016). From the results of these studies, some of these interactions may be generalizable and predictable (e.g. based on plant traits; Bullock *et al.* 2018). For example, vehicles were observed to disperse seeds in a directional manner in Berlin, in which seed traps near outbound lanes tended to have native seeds and exotic non-crop seeds, while inbound lanes tended to have exotic crops (von der Lippe and Kowarik 2008).

Recent empirical advances can aid a mechanistic understanding of seed dispersal and the development of mechanistic models described above (e.g. Nathan *et al.* 2011b; Cortes and Uriarte 2012). These advances include detailed data on seed movement (e.g. stable isotopes: Carlo *et al.* 2009; radio transmitters: Hirsch *et al.* 2012; DNA barcoding: González-Varo *et al.* 2014), animal movement (e.g. Movebank: Kranstauber *et al.* 2011; integrating GPS tracking with remote sensing: Kays *et al.* 2015; animal-borne sensors: Wilmers *et al.* 2015) and the abiotic environment (Baldocchi *et al.* 2001; Davies and Asner 2014). For example, using telemetric thread tags (Hirsch *et al.* 2012), Jansen *et al.* (2012) found that secondary dispersers have a greater role in LDD than previously thought. In addition, future research can link trait data (e.g. TRY: Kattge *et al.* 2011; D^3^: Hintze *et al.* 2013; KEW: Royal Botanic Gardens Kew 2016) to dispersal processes to help reduce complexity of interactions and models (Aslan *et al.* 2019). The quantity of data relevant for dispersal is increasing into the realm of big data (Allan *et al.* 2018), and rapid access is eased through curated repositories. These repositories can be used to improve our ability to incorporate intraspecific variation in seed dispersal (Schupp *et al.* 2019), which can have important consequences for plant populations, communities and evolution (Snell *et al.* 2019; Schreiber and Beckman 2020). While current advances allow us to study dispersal vector characteristics at very fine spatial and temporal resolution, the question remains whether this also captures variation among and within populations and species.

### Long-distance dispersal

Long-distance dispersal—often a rare event—is critical to the spread of populations (Ferrandino 1993; Kot *et al.* 1996; Hastings *et al.* 2005). Advances have been made in operationalizing the concept of LDD, but challenges remain concerning how to measure these rare events. Jordano (2017) recently introduced operative definitions of LDD using a demographic perspective in which propagules can contribute to LDD by expanding a species’ range when they colonize new areas outside of the source population or disperse away from close relatives outside of the genetic neighbourhood in which parents mate. He identified three types of LDD: (i) LDD within the genetic neighbourhood, (ii) short-distance dispersal outside of the genetic neighbourhood and (iii) strict-sense LDD (i.e. LDD outside the genetic neighbourhood). The question remains: once operationalized, how do we measure such rare events? This remains a major challenge in empirical ecology, but perspectives from population genetics, statistics, and physics are improving our ability to empirically measure the importance of LDD in gene flow and species distributions.

Genetic analysis of populations can link individuals to their source populations and has been a useful tool for understanding the importance of rare, long-distance events in colonizing new areas. Using genetic information from 25 species, Alsos and colleagues found that multiple dispersal events from several source regions contributed to post-glacial colonization of five islands in the Arctic; source regions were 280 to >3000 km away and were frequently not the closest ones—suggesting a greater role for deterministic rather than stochastic factors resulting in LDD (Alsos *et al.* 2007, 2015). Landscape genetics reveal that multiple LDD events were also responsible for mountain hemlock colonization on Alaska’s Kenai Peninsula (USA) following Pleistocene glaciation (Johnson *et al.* 2017). Using molecular techniques, observations of fruit consumption, and data from seed traps and faeces, Jordano *et al.* (2007) were able to quantify the contribution of different dispersal vectors to LDD for the mahaleb cherry (*Prunus mahaleb*) and found that LDD of this plant species is driven by a small subset of large frugivores. Citizen science projects can also shed light on the extent and magnitude of LDD events. For example, in Sweden, Auffret and Cousins (2013) found that humans dispersed meadow species, especially those with hooked or appendaged seeds and persistent seed banks, from 1.3 to 110 km.

Extreme value theory, introduced by Gumbel (1958), can be applied to dispersal distances obtained from molecular tools, tracking dispersal vector movement and censored data (e.g. the maximum observed distance moved from a fruiting tree) to estimate the frequency and extent of rare dispersal events. This statistical technique has been widely used in other disciplines such as climatology, hydrology, engineering, insurance, finance and, more recently, ecology to estimate the frequency and extent of rare events, for example, the return interval of large floods (e.g. Gaines and Denny 1993; Gutschick and BassiriRad 2003). Extreme value statistics have been applied to the study of plant dispersal only very recently, likely due to limited data in the past (García *et al.* 2017). Using data on seed dispersal distances obtained from genetic analyses of the vertebrate-dispersed mahaleb cherry, García *et al.* (2017) found that seeds could be dispersed outside of the focal population with low, but non-zero probability (Pr(*X* ≥ 1 km) = 0.10, Pr(*X* ≥ 5 km) = 4 × 10^−4^ and Pr(*X* ≥ 10 km) = 7 × 10^−5^). Extreme value statistics can give insight into invasions, the loss of dispersal services or the likelihood of populations tracking suitable habitat (García *et al.* 2017), and may therefore be useful for generalizing across systems. However, these methods are phenomenological models fit to existing data and assume stationarity, while extreme value statistics are likely to shift under global change.

Physics can contribute insights on measuring rare events mechanistically, which can help in predicting LDD under global change. In nature, there are two types of rare events: (i) discrete and uncorrelated, such as an unusually long pause between two consecutive events in a Poisson process, and (ii) a sequence of cumulative rare events. Long-distance dispersal by animals is most likely of the first type. Long-distance dispersal by wind is most likely of the second type, as an unlikely sequence of turbulent events sustain the seed in the air for an unlikely long period of time (all the while being pushed by wind in the direction parallel to the ground). Physicists have developed a powerful approach for understanding the statistics and dynamics of a sequence of cumulative rare events. A rare chain of events connects the initial and final state. In the case of a LDD event, the initial and final states would be the source and destination locations of a propagule. An event would be considered rare, if the dispersal event was much longer than a typical dispersal event (quantified, for instance, by the standard deviation of dispersal distances). The key insight behind this approach is that a very unlikely chain of events (e.g. a dispersal event that is much longer than a typical dispersal event) unfolds along an essentially deterministic realization—the least unlikely out of all unlikely realizations connecting these initial and final states. The more unlikely the chain of events, the more it will be dominated by this least unlikely realization (or chain of events). Deviations around this ‘optimal path’ quickly decrease in probability, even though the probability of this least unlikely path is also small (given some fixed waiting time). The Wentzel, Kramers, Brillouin (WKB) theory—originally developed for calculating the rates of (rare) tunnelling events in Quantum Mechanics—has found applications in fields as diverse as population biology and epidemiology in recent years (Ovaskainen and Meerson 2010). This WKB theory is the method by which an optimal path (or trajectory) can be found, and the probability of a rare event can be evaluated. Given the properties of noise (e.g. its correlation function), the method gives a certain cost function that measures the relative probability of any one path. Minimizing this cost function over the functional space of paths gives the optimal path, and the cost function along the optimal path gives the dominant contribution to the probability of a rare event. Attempts are currently under way to adapt this theory to hydrodynamics (Laurie and Bouchet 2015; Bouchet *et al.* 2018), overcoming challenges imposed by the high dimensionality of dynamics involved. Ovaskainen and Meerson (2010) provide both an accessible exposition of this technique and its biological applications, and a clear explanation of challenges of applying these ideas in complex situations.

### Scaling from individual seed movements to population-level patterns

Both generalizing and predicting population-level patterns of seed dispersal from the movements of individual seeds require relevant advances in data, mathematics and computation. First, it requires a detailed understanding of the mechanisms of the focal system as introduced above and the natural histories of the relevant players. Second, it requires mathematical and computational advances that efficiently scale from interactions occurring over short time scales (on the order of minutes) and spatial scales (on the order of mm–cm) to patterns emerging over months to years across landscapes and regions. Modeling population-level patterns of the movement of individual seeds in response to the action, behaviour and movement of dispersal vectors can be computationally expensive and require extensive data for calibration. There are only a few plant species for which complex, mechanistic models have been developed and for which enough data exist to parameterize these models to predict population-level spatial patterns of seed dispersal from individual seed movements (Nathan *et al.* 2011b; Cortes and Uriarte 2012). These models are better-developed for ballistic- and wind-dispersed plants than animal-dispersed plants (Nathan *et al.* 2011b; Cortes and Uriarte 2012), in large part because of the complexity of animal behaviour and movement (Zwolak 2018). However, the field of animal movement ecology has advanced tremendously over the last decades with tracking and analytical methods constantly improving (Börger 2016). Collaborating more closely with animal movement researchers opens up new opportunities for developing improved models of animal-dispersed plants.

To scale from individual movements to population-level patterns, we can approximate complex mechanistic models with models that make simplifying assumptions. One multi-scale mathematical approach is to begin with random walks of individuals and use various approximations to arrive at diffusion or transport equations that describe the collective movement of individuals (Turchin 1998; Codling *et al.* 2008). Coupled with functions of seed retention time (Bullock *et al.* 2011), these approximations can describe seed dispersal by animals and give important insight into how variability in retention times (in animal guts or externally) influences LDD (Guttal *et al.* 2011). A fluid dynamics approach used by physicists, hydrologists and atmospheric modellers can help overcome challenges in scaling from the local interactions of a seed with the physical dynamics of wind and water to large-scale dispersal patterns. Under certain assumptions, the effect of wind on the dispersal kernel and LDD can be approximated using analytical mathematical results, such as the Wald analytical LDD model (e.g. Katul *et al.* 2005). The empirical, analytical and numerical methods for the treatment of abiotic dispersal in heterogeneous landscapes require further development in future research (Brinkman 1949; Bohrer *et al.* 2008; Nathan *et al.* 2011b; Katul and Poggi 2012; Trakhtenbrot *et al.* 2014). Advances have been made on this front by Powell and Zimmermann (2004), who developed an analytical solution to approximate the migration of plants dispersed by animals based on the theory of homogenization, which could be extended to abiotically dispersed plants. Through this technique, Powell and Zimmermann (2004) incorporated caching activity by harvester ants for wild ginger (*Asarum canadense*), by Blue Jays (*Cyanocitta cristata*) for oaks (*Quercus*) and by Clark’s nutcrackers (*Nucifraga columbiana*) for whitebark pine (*Pinus albicaulis*), and were able to predict migration rates of trees that matched the paleo-record, except in the case of the Holocene migration of wild ginger.

Future research can advance mechanistic dispersal kernels and predictive seed dispersal ecology. First, dispersal kernels can better incorporate interactions between dispersal vectors and individual seeds, which tend to occur across multiple spatial and temporal scales. For example, different dispersal mechanisms can be incorporated mathematically into the dispersal kernel, which gives the long-term limit after all seeds land, to evaluate the effects of different vectors quantitatively using methods from applied mathematics (Rogers *et al.* 2019). Second, dispersal kernels should account for non-stationarity in driving factors and depend on the environment. That way, kernels can change with time, space and shifts in the environment, important for predicting dispersal in novel landscapes. Standardized data initiatives provide a valuable means for evaluating the magnitude and causes of non-stationarity across space and time. Third, integrating multiple dispersal vectors, non-stationary dispersal kernels, and improved and standardized dispersal vector monitoring and data collection with process-based models will allow predicting the spatiotemporal distribution of seeds of entire populations across the landscape.

## Gap 2: Understanding the demographic consequences of seed dispersal

So far, we have discussed how to understand seed dispersal as a process. To understand the importance of seed dispersal for the dynamics of a population over multiple generations, we need to understand how this process interacts with stages across a plant’s entire life cycle, from seed production through juvenile and adult survival and growth. Prediction of the demographic consequences of seed dispersal remains a large challenge due to the context dependence of seed dispersal (Schupp *et al.* 2010), heterogeneity of the environment (Nathan *et al.* 2011a), the long lifespans of many adult plants and interdependent processes occurring over multiple spatial and temporal scales (Mokany *et al.* 2014). A promising path forward is integrating the Seed Dispersal Effectiveness Framework (Schupp 1993; Schupp *et al.* 2010) with advances in mathematical and computational methods (e.g. Godinez-Alvarez and Jordano 2007; Cortes and Uriarte 2012). The Seed Dispersal Effectiveness Framework—an important progression in embracing the context dependence of seed dispersal and moving towards an ability to generalize across systems—provides a roadmap for evaluating the contribution of each dispersal vector to the production of a new adult by evaluating the *quantity* of seeds dispersed and *quality* of seed dispersal in different contexts (Schupp 1993; Schupp *et al.* 2010). This information can be incorporated into process-based dynamic models of populations to examine the influence of dispersal compared to other life-history stages on the growth and spread of populations over multiple generations as discussed below.

### Local population dynamics

To evaluate the role of seed dispersal in population dynamics, we need to explicitly integrate over critical determinants of seed dispersal effectiveness (Schupp *et al.* 2010), including pre-dispersal, dispersal and post-dispersal processes operating across different life stages. Hitherto, the Seed Dispersal Effectiveness Framework has mostly been applied to single species (but see Donoso *et al.* 2016; Fricke *et al.* 2018), but Aslan *et al.* (2019) outline an approach to generalize across functional groups. Such attempts at generalizing seed dispersal effectiveness across species and systems are necessary because empirical data for operationalizing the Seed Dispersal Effectiveness Framework are still scarce (but see Simmons *et al.* 2018). For example, data on dispersal and its delayed consequences for plant survival and growth are limited in temporal scale, following seeds for only a few years (Clark *et al.* 1999a; Howe and Miriti 2004), and are highly species-specific, with data amount and quality varying widely among plant species and interacting species that influence plant dispersal, growth and survival (Agrawal *et al.* 2007). Additionally, for long-lived plants such as trees, we are limited to collecting data on the early stages of recruitment. As a result, it is unclear how variation in dispersal and heterogeneity in the seedscape across space and time will influence later stages of recruitment. Population models constitute an alternative approach, and parameterizing population models with observational and experimental data on the effectiveness of different dispersal vectors and their deposition in varying seedscapes helps elucidate the role of dispersal in the demographic process (e.g. Brodie *et al.* 2009b). Local population dynamics can be modelled to assume a range of biological complexity (reviewed in Jongejans *et al.* 2008). Such models include unstructured population models (e.g. exponential growth), structured population models that include stages or ages (e.g. matrix population models [MPMs]; Caswell 2001), spatially explicit IBMs (e.g. Adler and Muller-Landau 2005; Beckman *et al.* 2012) and dynamic vegetation models (DVMs; Snell *et al.* 2014).

 An extremely powerful set of analytical tools have been developed for both MPMs and integral projection models (IPMs) to predict population growth rate, stable stage distribution and sensitivity to small perturbations in the model parameters (Caswell 2001; Ellner and Rees 2006). These analytical models rely on the law of large numbers, and thus model mean populations that encounter each other in proportion to their average abundance (i.e. a mean-field assumption), generally assume homogeneous environments, and provide asymptotic results. Such structured population models are useful to examine different hypotheses of how present conditions influence populations by examining population projections (Caswell 2001). We can use these models to analyse the population growth rate and its sensitivity under different dispersal scenarios (e.g. no dispersal, one dispersal vector, a community of dispersal vectors), and this has improved our understanding of whether and under what conditions dispersal is important for a particular species and our ability to predict the consequences of shifting the community of dispersal vectors. For example, Godinez-Alvarez and Jordano (2007) proposed integrating the Seed Dispersal Effectiveness Framework with MPMs to evaluate the influence of dispersal vectors on the dynamics of plant populations. By building the projection matrix based on the quantity and quality of dispersal by one bat and three bird seed dispersers, Godinez-Alvarez and Jordano (2007) found that dispersal vector identity influenced population growth rates of the cactus *Neobuxbaumia tetetzo*. In MPMs, plants are categorized in discrete stages by size or life-history stage (e.g. seed, seedling, juvenile, adult), which is more appropriate for plants as they can remain in the same stage for multiple years and/or have unknown or difficult-to-measure ages. Integral projection models can accommodate both discrete and continuous descriptions of fecundity, survival and growth based on size and age (Easterling *et al.* 2000), which is especially important for long-lived species as individual variation within stages can influence population dynamics (Zuidema *et al.* 2010). The dynamics of transients, important in the conservation and management of populations, can also be analysed (Caswell 2006). For example, Elwood *et al.* (2018) showed that scatter-hoarders can have significant effects on both short- and long-term population dynamics of American Chestnut (*Castanea dentata*). An important advance in analysing dynamics of structured population models is the development of tools to examine consequences of random variation in vital rates (i.e. stochastic demography; Boyce *et al.* 2006). This stochastic variation affects estimates of population growth, persistence and resilience compared to deterministic versions of structured population models (Boyce *et al.* 2006). As anthropogenic pressures can increase or decrease environmental variability, the implications of this variation for demography should be carefully considered (e.g. Snell *et al.* 2019). In addition, future research can explicitly include post-dispersal mechanisms, such as competition, mortality due to natural enemies and microsite suitability for growth (Howe and Miriti 2004) into these suite of population models to increase their capability of predicting dynamics in response to novel conditions.

To explicitly incorporate post-dispersal processes, researchers could use a systems approach to examine the influence of dispersal by animals on local plant dynamics. Cortes and Uriarte (2012) proposed integrating the Seed Dispersal Effectiveness Framework with the movement ecology paradigm developed by Nathan and colleagues (2008) that combines internal states, motion and navigation capacities of individuals with external factors to study movement. This could be done with IBMs (Grimm and Railsback 2005) or DVMs (Snell *et al.* 2014). For example, Loayza and Knight (2010) used an IBM parameterized by field studies on seed dispersal movement and the quantitative and qualitative components of seed dispersal effectiveness for two bird dispersers of the tree *Guettarda viburnoides* in a forest-savanna mosaic in Bolivia. Their model predicted that dispersal by Purplish Jays (*Cyanocorax cyanomelas*; pulp consumers which frequently dropped seeds) increased population growth due to a positive impact of seed handling and an increased likelihood of reaching suitable habitat (woody patches), whereas dispersal by Chestnut-eared Aracaris (*Pteroglossus castanotis*; ‘legitimate’ seed dispersers that swallow the fruit whole and pass the endocarp intact) decreased population growth, due to dispersal to unsuitable habitats (forest islands). Dynamic vegetation models include demographic, ecological and physiological processes as well as biotic interactions (i.e. competition) and range from models that simulate forest dynamics through growth and mortality of individual trees to models that simulate biogeochemical cycles and vegetation distributions through plant functional types (Snell *et al.* 2014). However, only a few DVMs currently include seed dispersal (e.g. Sato *et al.* 2007; Snell 2014; Snell and Cowling 2015; Lehsten *et al.* 2019), and none yet includes the level of detail outlined here. Dynamic vegetation models with more realistic seed dispersal processes can capture interactions in novel non-analogue environments, useful for predicting population dynamics when interspecific interactions and demographic processes shift.

Data requirements for population models can come from long-term observational studies, manipulative or accidental experiments (e.g. systems that have lost dispersers as a result of global change; HilleRisLambers *et al.* 2013), or combinations thereof. Data on the dispersal process as discussed above, including the action/activity, occurrence, abundance and movement patterns of dispersal vectors, inform potential deposition sites of different dispersal vectors. Long-term data from censusing give information on survival, growth and recruitment through time and space (i.e. the quality of seed dispersal), necessary for long-lived species. Field and greenhouse experiments can provide detailed information on the suitability of deposition sites for plant recruitment. Seed addition experiments in different habitats can be used to quantify how the action/movement of different seed dispersal vectors influence recruitment in different microsite conditions (Turnbull *et al.* 2000; Clark *et al.* 2007). To evaluate the influence of interspecific interactions within deposition sites, controlled greenhouse and field studies can exclude mycorrhizal and nurse–plant associations to measure the effect of these changes on plant growth and nutrient exchange (van der Heijden 2010) or impose or simulate herbivory, seed predation, pathogen attack or parasitism to measure growth rates and other fitness correlates in the presence of natural enemies (e.g. Agrawal 1999). Accidental experiments enable researchers to functionally manipulate dispersal or realistically simulate its absence and evaluate the impact across all life-history stages, not just those that are most tractable for experiments (e.g. seeds, small seedlings). For example, Brodie *et al.* (2009a) predicted a decline in population growth rate of the canopy tree *Choerospondias axillairs* in over-hunted forests using a combination of accidental experiments, manipulative seed germination experiments, and population matrix models. How population growth rates differ depending on the quality of seed dispersal across different life-history stages and habitats can be investigated within population models using life table response experiment (LTRE) analyses (Caswell 2001). A LTRE (term introduced by Caswell 1989) compares vital rates under different experimental or observational conditions. A LTRE analysis examines differences in a demographic summary statistic derived from these vitals rates, such as population growth rate, across the study conditions. This is done by decomposing differences in the demographic summary statistic into contributions from the differences in vital rates across study conditions (Caswell 1989, 2001). For example, Loayza and Knight (2010) used a LTRE analysis to compare population growth rates of the tree *G. viburnoides* between two habitats in which seeds are deposited by different bird seed dispersers. They decomposed the difference in predicted population growth due to contributions from the differences in seedling growth, small tree growth and adult tree fecundity (Loayza and Knight 2010).

### Population spread

Dispersal and population spread are at the centre of a fundamental question in global change biology and invasion ecology (Clark *et al.* 1998; Pauchard and Shea 2006; Jongejans *et al.* 2008, 2011; Lockwood *et al.* 2013): if habitats change due to habitat destruction or climate change, will seed dispersal and population growth allow the plant population to track its suitable habitat? Or if a plant species’ seeds are transported into a novel habitat, will seed dispersal and population growth allow the species to naturalize or even become invasive? Information on dispersal processes and demographic transitions from the Seed Dispersal Effectiveness Framework can aid the development of models to predict the spread of populations invading new areas and evaluate the relative importance of seed dispersal. Analytical approaches used to model population spread include *reaction-diffusion models* that combine continuous time population models with diffusion (i.e. population-level approximation of random walks as discussed in *Scaling from individual seed movements to population-level patterns*), which are widely and successfully used in spatial ecology (Okubo and Levin 2002; Cantrell and Cosner 2004). Their discrete time analogues, *integrodifference equations (IDEs)*, offer several appealing features for modeling plant populations. Integrodifference equations can incorporate discrete stage structure (Neubert and Caswell 2000) and more closely represent seasonality in natural systems. They also offer greater flexibility in describing dispersal events via redistribution kernels (or probability density functions for seed shadows) (Kot and Schaefer 1986). As discussed in the previous section, systems approaches can also be used to model population spread rates, which assume discrete interacting individuals. Santini *et al.* (2016) found that IBMs predicted slower spread rates of mammals compared to an IDE, most likely due to the inherent stochasticity in IBMs. A functional perspective of seed dispersal effectiveness (Aslan *et al.* 2019) could help the incorporation of dispersal into DVMs to simulate range shifts of plants (Snell *et al.* 2014). For an overview of the types of models that integrate dispersal and demography, see Jongejans *et al.* (2008).

Integrodifference equations (IDEs) have been used to examine the spread of invading organisms (Kot *et al.* 1996; Hastings *et al.* 2005; Skarpaas and Shea 2007) and the influence of climate change on shifts in species ranges (Zhou and Kot 2011; Harsch *et al.* 2014). Exponentially bounded kernels result in constant speed of population spread/invasion in integrodifference equations. However, fat-tailed kernels, such as the bivariate version of Student’s *t*-distribution that fits many dispersal vector–plant combinations (Clark *et al.* 1999b), may lead to accelerating invasion speeds (Kot *et al.* 1996). Clark *et al.* (2001) developed an alternative approach to estimate finite spread rates using the expected velocity for the location of the furthest-forward individual. Using this method, they found slower spread rates than predicted by analytical models, and these slower rates were in line with paleorecords (Clark *et al.* 2001). Mechanistic models for wind-dispersed species that incorporate dispersal and demography have been used to determine causes of variation and predict spread rates in response to climate-mediated changes in dispersal (Nathan *et al.* 2011a; Bullock *et al.* 2012; Teller *et al.* 2016). Life table response experiment analyses (introduced in the previous section) of IDEs can determine the contributions of differences in demography and dispersal to differences in spread rates across populations as was done for both inter- and intraspecific bird populations by Caswell *et al.* (2003) and different management scenarios of the annual herb *Rhinanthus minor* (Bullock *et al.* 2008). By integrating seed dispersal effectiveness with LTRE analysis, researchers can examine how different dispersers influence population spread rates through their effects on demography and dispersal or how changes in vital rates and seed dispersal due to global change could influence population spread rates. Recent advances provide new opportunities to understand the influence of dispersal processes on population spread. Mathematicians have developed promising approaches to incorporate individual variation (spatial IPMs; Jongejans *et al.* 2011), fragmented landscapes (i.e. reaction-diffusion models: Maciel and Lutscher 2013; integrodifference equations: Gilbert *et al.* 2014), stochasticity (Caswell *et al.* 2011) and temporally variable environments (Caswell *et al.* 2011; Schreiber and Ryan 2011; Ellner and Schreiber 2012). A good description of a variety of methods for calculating discrete-time invasion rates from data is available in Lewis *et al.* (2006).

Empirical advances to measure spread (e.g. remote sensing via unmanned aerial vehicles and telemetry) can be combined with models to elucidate important dispersal vectors. For example, Vellend *et al.* (2006) estimated migration rates for *Trillium grandiflorum* using an IDE parameterized with data on deer movements from telemetry and gut passage to describe dispersal and demographic transitions under different levels of herbivory, and these estimates were much faster than previous estimates based on ant dispersal. In addition, vehicles can disperse seeds long distances and facilitate the spread of invasive species that can disrupt land management and ecosystem function of natural plant communities. For example, vehicles aided the spread of cheatgrass, which has overtaken sagebrush in the western arid regions of the USA (Strickland *et al.* 2015)—and this has implications for cattle grazing and water storage. Future research should further develop approaches to determine if and when it is necessary to consider LDD in the context of population spread (Kot *et al.* 1996). For determining spread rates of populations, Neubert and Caswell (2000) suggested that data on the distances dispersed by seeds are more important than knowing the proportion of seeds dispersed at long distances—as long as this proportion is small—and that it is more feasible to measure the distance travelled by LDD vectors than the proportion dispersed by each vector that results in different dispersal kernels.

## Recommendations for future research

Moving towards a mechanistic and predictive understanding of the movement of seeds and the demographic consequences of this movement requires collaboration across a large group of scientists working at different scales, in different bioregions, using a wide arsenal of tools. At present, seed dispersal research is carried out by researchers from an array of subdisciplines with diverse but poorly aligned goals and approaches. Disparate literature bodies investigate seed dispersal from ecological, mathematical, theoretical, computational, statistical, genetic, physical and evolutionary angles. While each subdiscipline can contribute insights into particular aspects of seed dispersal, no single disciplinary method or conceptual framework can independently close the loop on seed dispersal and its contributions to plant populations. We provide recommendations for future research focusing on strategies to accommodate diverse but potentially limited data.

### Collate existing, disparate data sets

The highly context-dependent nature of empirical data and limited knowledge of dispersal and its consequences for plant fitness impede our ability to generalize and predict response of plants under global change. However, there is a wealth of available knowledge that has not yet been synthesized for analysis. Rich new data sets are currently emerging in ecology as a result of advances in remote sensing data (Kerr and Ostrovsky 2003; Pettorelli *et al.* 2014), environmental sensor data (Rundel *et al.* 2009; Wilmers *et al.* 2015), long-term data from research sites such as Long-term Ecological Research (LTER) and Forest Global Earth Observatory (ForestGEO; Anderson-Teixeira *et al.* 2015), emergence of new large collaborative networks (e.g. Templ *et al.* 2018; USA National Phenology Network 2018) and globally distributed experiments (e.g. Nutrient Network; Borer *et al.* 2014), and increased digital availability of data. Data sources include publicly available data sets, including data on dispersal distances (e.g. Tamme *et al.* 2014; Bullock *et al*. 2017), traits (e.g. Kleyer *et al.* 2008; Kattge *et al.* 2011; Royal Botanic Gardens Kew 2016), networks (e.g. Pigot *et al.* 2016), demography (e.g. Salguero-Gómez *et al.* 2015), plant phylogenies (useful for generalization based on cross-species comparisons and understanding the evolutionary implications of seed dispersal; Zanne *et al.* 2013) and species distributions (e.g. Enquist *et al.* 2016), and unpublished data sets (seed dispersal networks, dispersal kernels, spatial dispersal data, movement data, etc.). There is also an abundance of existing data within the gray and white literature, including data from conservation areas and government organizations, in a variety of languages. Automated text analysis as used in the social sciences (e.g. Wilkerson and Casas 2017) can identify documents with relevant data in multiple languages. Currently, a repository for data on dispersal processes is lacking and requires appropriate cyberinfrastructure to assimilate large quantities of disparate data into models. Existing cyberinfrastructure, such as the National Science Foundation-funded CyVerse developed for the life sciences, is one option. CyVerse allows for flexible storage of heterogeneous data and is able to interface with existing repositories that house relevant data (Goff *et al.* 2011; Merchant *et al.* 2016). The time is ripe for creating a repository for dispersal data for synthesis and analysis. These data can be linked to existing available data sets to close the two knowledge gaps discussed above and improve our ability to generalize across systems and predict outcomes for specific systems.

### Use novel statistical techniques to integrate disparate data with process-based models

Differences in model structure and parameterization based on limited data can create large uncertainties in model predictions (Hartig *et al.* 2012) and necessitates systematic examination of dispersal mechanisms as well as high-resolution data (Cortes and Uriarte 2012; Mokany *et al.* 2014). We can take advantage of systematic reviews and statistical approaches, such as meta-analyses (e.g. Markl *et al.* 2012), inverse modeling (e.g. Ribbens *et al.* 1994) and imputation methods (e.g. Santini *et al.* 2016), to integrate the growing body of available data with process-based models. Systematic reviews and meta-analyses can help identify processes that require model development, as well as parameter ranges for these models. Recent statistical advances in merging process-based models with Bayesian or approximate Bayesian methods can reduce uncertainty by incorporating different types of data (Hartig *et al.* 2011, 2012), facilitating identification of relevant processes by better utilizing existing data, a major advantage of modern statistics and computing that has not yet been exploited. For example, approximate Bayesian approaches (e.g. ABC) enable one to infer parameters from a variety of process-based models including stochastic individual-based simulation models, which cannot be informed by statistical theory such as maximum likelihood or Bayesian methods because their likelihood functions cannot be explicitly calculated. In addition, new methods are continuing to be developed to accommodate sparse data and fill gaps in trait data (e.g. Swenson 2014; Schrodt *et al.* 2015; Santini *et al.* 2016).

### Scale from the movement of individual seeds to population-level patterns of dispersal and recruitment using analytical approximations

Using analytical models developed from empirical data, we can explore alternative hypotheses regarding dispersal that can be tested in the field, make broadly applicable predictions that can be evaluated across systems and explore sensitivity to parameters (important when data are limited; Bullock *et al.* 2012). Results from these empirical studies enable the refinement of theoretical models. In cases where it would be infeasible or unethical to use empirical experimentation at the scales necessary to explore population dynamics, models can be used to evaluate competing hypotheses. Approximations require less data for parameterization and are efficient, and thus can help inform pressing management issues (Travis *et al.* 2011). Finally, these approximations can be included as submodels of more complex simulation models to reduce their complexity and data requirements and predict consequences of dispersal at larger organization, spatial or temporal scales.

### Conduct sensitivity analyses of models to determine sufficiency of available data

Developing process-based models can guide effective data collection by determining the sensitivity of models to variation in parameters or structure (Milner-Gulland and Shea 2017). For example, parameters that are identified as being disproportionally important for determining plant responses will require more detailed data collection (e.g. Nathan *et al.* 2011b; Mokany *et al.* 2014). We can examine whether missing data or poorly parameterized values influence model output or produce contradictory patterns. Models can guide the choice of empirical sampling designs and appropriate statistical models by evaluating the sensitivity of results to different sampling designs and statistical models (virtual ecologist approach; Skarpaas *et al.* 2005; Zurell *et al.* 2010). Finally, this process will guide the development of methods and protocols for standardized data collection that can be included in both existing and new long-term studies. Standardized data collection efforts informed by theory will facilitate cross-site comparisons in both data analysis and model outputs, can help evaluate model predictions and will facilitate the investigation of future questions in seed dispersal ecology.

### Create coordinated research networks and standardized data collection protocols to fill remaining data gaps

We encourage researchers to coordinate research activities and utilize a variety of empirical methods (e.g. censuses, seed traps, genetics, radio-tracking, remote sensing, etc.) to study a diversity of seed dispersal vectors and plant growth forms (woody plants, herbaceous plants, grasses, etc.) building upon existing standardized data collection protocols and global networks (e.g. Borer *et al.* 2014; Anderson-Teixeira *et al.* 2015; Saatkamp *et al.* 2019). A summary of data needs as identified by the participants of the Seed Dispersal Workshop is provided in **Supporting Information —File S1**. Empirical ecologists are able to generate important case study data on local processes occurring over short time periods that can serve as model systems for testing theory. We can use theory to examine whether and how information from case studies can be generalized across systems and extrapolated to larger organizational, spatial and temporal scales. In addition, coordinating and standardizing data collections can help overcome shortcomings in empirical studies to increase the number of focal species and the spatial and temporal scope. Often empirical ecologists are geographically scattered, and researchers working in tropical vs. temperate systems or Old World vs. New World systems are largely segregated—publishing in different journals and attending different conferences. Therefore, increasing international collaborations and global integration across regions will be necessary to enable generalization to ecosystems worldwide. Based on participant experiences described at the Seed Dispersal Workshop, there seems to be little communication among researchers studying abiotic vs. biotic dispersal vectors or among researchers working on biotic dispersal vectors, that is researchers working on endozoochoric, epizoochoric and seed-caching organisms. Linking these perspectives may advance our understanding of the importance of different dispersal vectors. In addition, we propose that closer collaborations among ecologists, mathematicians, hydrologists, atmospheric modellers and physicists exploring the movement of animals, water and wind will bring important insights to these efforts.

### Predict consequences of dispersal over larger organization and spatiotemporal scales

System-specific forecasts will require the development and application of novel analytical and efficient computational methods for models. Computational models based on dispersal theory and parameterized with system-specific data hold promise for evaluating the importance of dispersal within ecosystems. Such generalizations may elucidate the qualitative and quantitative effects of species-specific dispersal kernels and disperser loss on plant populations. Collaboration and information sharing between empiricists, mathematicians, modellers and theoreticians may help address this challenge, by directing empirical data collection to efficiently address model parameter needs and by helping ecological modellers to incorporate relevant variables as they develop increasingly mechanistic models. These models can be evaluated with future empirical studies.

## Conclusions

To tackle the complexity and context dependency of seed dispersal, we urge a better integration of empirical and theoretical approaches. This requires enhanced communication and collaboration across researchers in different disciplines, across geographic locations, and studying different aspects of plant life histories and environmental conditions that influence dispersal and demography. Existing models need to be further developed and refined to evaluate the role of dispersal on population persistence and spread; better predict extinction risk of species; and evaluate conservation and management strategies. Synthesis of data on dispersal processes, seed dispersal effectiveness across multiple life-history stages and demography represents an opportunity to develop theory for generalization across systems and to identify relevant processes that require model development and data collection for system-specific predictions.

## Supporting Information

The following additional information is available in the online version of this article—

File S1. Summary of data needs.

plz048_suppl_Supplementary_Material

## Sources of Funding

Ideas for this manuscript initiated during the Seed Dispersal Workshop held in May 2016 at the Socio-Environmental Synthesis Center in Annapolis, MD and supported by the US National Science Foundation Grant DEB-1548194 to N.G.B. and the National Socio-Environmental Synthesis Center under the US National Science Foundation Grant DBI-1052875. D.Z. received funding from the Swiss National Science Foundation (SNF, grant: PZ00P3_168136/1) and from the German Science Foundation (DFG, grant: ZU 361/1-1).

## Contributions by the Authors

N.G.B. led the development of the concepts, writing and revising of the manuscript with input from C.E.A. and H.S.R. All authors contributed to the development of concepts and are listed in order of contribution and alphabetical order within each level of contribution.

## Conflict of Interest

None declared.
