# Supplementary material for: Advancing an interdisciplinary framework to study seed dispersal ecology
Source: AoB Plants. 2019 Aug 19;12(2):plz048. doi: 10.1093/aobpla/plz048 (PMC7179845; doi:10.1093/aobpla/plz048)
Supplement: plz048_suppl_Supplementary_Material [file plz048_suppl_supplementary_material.pdf]

SUMMARY OF DATA NEEDS

Below is a summary of data needs as identified by participants of the Seed Dispersal Workshop (May 2016). While many of these data are collected independently, we lack comprehensive data on dispersal and demography in the same system and for the same taxa. We also highlight existing available data that need to be combined with dispersal data. This is not a comprehensive list of data needs but were those identified as important gaps to be filled.

Movement of Seeds

Animal-dispersed species

Adhesion rates
Disperser residence times in different habitats
Handling times
Gut passage time - more of a continuous evaluation
Animal Movement across different habitats

- mark-release-recapture
- telemetry data
- *Existing*: Global data archive of animal tracking data with 811 taxa, 1 billion locations, 1.4 billion non-location events in Movebank (Kranstauber *et al.* 2011, Wikelski and Kays 2018)

Animal movement linked to gut passage
Animal Traits

- *Existing*:
  - Traits for 214 extant mammal and 242 bird species of Madagascar (Razafindratsima *et al.* 2018)
  - Global database of species-level foraging attributes for 9993 and 5400 extant bird and mammal species (Wilman *et al.* 2014)
  - Global database of diet preferences of 5364 mammals (Kissling *et al.* 2014)
  - Global database of traits, phylogeny, and range maps for 5831 known mammal species extant since the last interglacial, including current ranges and present natural ranges predicted in the absence of anthropogenic pressures (Faurby *et al.* 2018)
  - Global database of bird and mammal traits (volancy, diurnal/nocturnal, clutch size, body mass, habitat breadth) for all bird and mammal species,

with values derived (and missing values phylogenetically imputed) from other existing databases (Cooke *et al.* 2019)
    - Global database of bird bill morphology from 3D scans of museum specimens, including landmark data. Initial dataset (roughly 1 species per genus) available at http://data.nhm.ac.uk/dataset/markmybird and species-level coverage expected soon (Cooney *et al.* 2017)

Time/space budgets of animal

Animal foraging, including seasonality of animal foraging

Demography

- *Existing:* 1927 matrix population models from 405 species in COMADRE (Salguero-Gómez *et al.* 2016, COMADRE Animal Matrix Database 2018)

Interaction Networks

Multi-year interaction networks

Interchangeability of dispersers and interactions between them

Beta diversity of interaction networks

Abundance of organisms out there (so we can understand opportunism vs. trait-based drivers of network structure)

Existing:

- Web resources and interfaces with seed dispersal interactions and other interaction types:
    - Interaction Web Database (https://www.nceas.ucsb.edu/interactionweb/)
    - Web of Life (http://www.web-of-life.es/)
    - Globi (Global Biotic Interactions; https://www.globalbioticinteractions.org/)
        - Most current and largest database of interactions. Although data from network studies are presented in this database, they are not presented as adjacency matrices as in the other resources. R package to access the data (Poelen *et al.* 2018), and an active platform to incorporate additional data via github (https://github.com/ropensci/rglobi), are also available.
- Studies that develop databases of seed dispersal network interactions compiled from multiple studies and that make these databases available in supplementary materials:
    - A database of networks involving fruit-eating neotropical bats (Mello *et al.* 2011a, b)

- A database of plant-frugivore interactions, including bird-plant interactions only, with substantial overlap with the Web of Life networks (Pigot *et al.* 2016).

Wind-dispersed species

Settling velocities
Wind speeds
Turbulence
Variances
Directions with seeds
Existing: Micrometeorological data available from the FLUXNET Network (http://fluxnet.ornl.gov/; http://fluxnet.fluxdata.org; Baldocchi *et al.* 2001)

Water-dispersed species

Flow speeds
Turbulence

Plant data

Presence/absence data

- *Existing:*
  - 206,241,288 observations from 485, 902 species Botanical Information and Ecology Network (Enquist *et al.* 2016, Enquist *et al.* 2018)
  - 1,038,689,568 occurrence data from over 1,000,000 species from the Global Biodiversity Information Facility (GBIF: The Global Biodiversity Information Facility 2018)

Fitted dispersal kernels

- *Existing*: Data available for 144 vascular plant species from 63 families (Bullock *et al.* 2017)

Spatial patterns of plants - subsample at smaller scales (more continuous)

- *Existing*: Forest Global Earth Observatory (ForestGEO; Anderson-Teixeira *et al.* 2015)

Seed rain relative to distance from conspecifics
Long distance dispersal

- *Existing*: Maximum dispersal distances available for 576 plant species from 102 families (Tamme *et al.* 2014)

Landscape genetics

Fruit/seed production (variability and environmental drivers of)

Plant Demography

Seed dispersal effectiveness

- quality of the microsite
- distance-dependent and density-dependent mortality

Demographic transition probability matrices for use in matrix models

- at low and high densities for population spread questions
- along climatic gradient
- *Existing*: 7024 matrix population models from 695 species (Salguero-Gómez *et al.* 2015, COMPADRE Plant Matrix Database 2018)

Fitted integral projection model (IPM) for population modeling

Datasets for IPM's over time

Plant traits

Fruit/seed trait information

- *Existing*:
    - KEW Royal Botanical Gardens Seed Information Database (Royal Botanic Gardens Kew 2016): Dispersal syndromes (25,050spp), seed weights (25,050spp), germination rates (12,424 spp)
    - LEDA Traitbase: 26 plant traits from *ca*. 3000 species of the Northwest European flora (https://uol.de/en/biology/landeco/research/projects/leda/; Kleyer *et al.* 2008)
    - TRY Plant Trait Database (148,000 plant taxa; Kattge *et al.* 2011)
    - FRUBASE: Fleshy fruit traits for more than 1000 plant species (Jordano 1995a, b)
    - Global database of fruit traits for 13,178 fleshy fruited plant species (Sinnott-Armstrong *et al.* 2018)
    - eFloras (online flora; Brach and Song 2006)
    - USDA PLANTS Database (http://plants.usda.gov)
    - Useful Tropical Plants Database contains information on the uses of thousands of plants grown in tropical regions (http://tropical.theferns.info)

Source plant traits

Changes in traits with climate

Population-level data

Population densities and spread over time

Environmental data

Weather conditions
Soil conditions (e.g. nutrient availability, moisture content)
Light availability

Existing: Environmental data from 81 field sites (47 terrestrial and 34 aquatic) in the United States from the National Ecological Observatory Network (National Ecological Observatory Network 2018)

References

Baldocchi D, Falge E, Gu L, Olson R, Hollinger D, Running S, Anthoni P, Bernhofer C, Davis K, Evans R, Fuentes J, Goldstein A, Katul G, Law B, Lee X, Malhi Y, Meyers T, Munger W, Oechel W, U KTP, Pilegaard K, Schmid HP, Valentini R, Verma S, Vesala T, Wilson K, Wofsy S. 2001. FLUXNET: A New Tool to Study the Temporal and Spatial Variability of Ecosystem-Scale Carbon Dioxide, Water Vapor, and Energy Flux Densities. *Bulletin of the American Meteorological Society* 82:2415-2434.

Brach AR, Song H. 2006. eFloras: New Directions for Online Floras Exemplified by the Flora of China Project. *Taxon* 55:188-192.

Bullock JM, González LM, Tamme R, Götzenberger L, White SM, Pärtel M, Hooftman DAP. 2017. A synthesis of empirical plant dispersal kernels. *Journal of Ecology* 105:6-19.

COMADRE Animal Matrix Database. Max Planck Institute for Demographic Research, Germany. Available at www.compadre-db.org. (data downloaded on November 2018, version 2.0.1).

COMPADRE Plant Matrix Database. Max Planck Institute for Demographic Research, Germany. Available at www.compadre-db.org. (data downloaded on November 2018, version 4.0.1).

Cooke RSC, Bates AE, Eigenbrod F. 2019. Global trade-offs of functional redundancy and functional dispersion for birds and mammals. *Global Ecology and Biogeography* 0.

Cooney CR, Bright JA, Capp EJR, Chira AM, Hughes EC, Moody CJA, Nouri LO, Varley ZK, Thomas GH. 2017. Mega-evolutionary dynamics of the adaptive radiation of birds. *Nature* 542:344.

Enquist BJ, Condit R, Peet RK, Boyle B, Schildhauer M, Dolins S, Thiers BM. *Botanical Information and Ecology Network*. Available at http://bien.nceas.ucsb.edu/bien/. (data downloaded on version BIEN 4.1).

Enquist BJ, Condit R, Peet RK, Schildhauer M, Thiers BM. 2016. Cyberinfrastructure for an integrated botanical information network to investigate the ecological impacts of global climate change on plant biodiversity. *PeerJ Preprints* 4:e2615v2.

Faurby S, Davis M, Pedersen RØ, Schowanek SD, Antonelli1 A, Svenning J-C. 2018. PHYLACINE 1.2: The Phylogenetic Atlas of Mammal Macroecology. *Ecology* 99:2626-2626.

GBIF: The Global Biodiversity Information Facility. *What is GBIF?* Available at https://www.gbif.org/what-is-gbif (data downloaded on November 2018).

Jordano P. 1995a. Angiosperm fleshy fruits and seed dispersers: A comparative analysis of adaptation and constraints in plant-animal interactions. *American Naturalist* 145:163-191.

Jordano P. 1995b. *Data from: Angiosperm fleshy fruits and seed dispersers: a comparative analysis of adaptation and constraints in plant-animal interactions*. Dryad Digital Repository.

Kattge J, Díaz S, Lavorel S, Prentice IC, Leadley P, Bönisch G, Garnier E, Westoby M, Reich PB, Wright IJ, Cornelissen JHC, Violle C, Harrison SP, Van Bodegom PM, Reichstein M, Enquist BJ, Soudzilovskaia NA, Ackerly DD, Anand M, Atkin O, Bahn M, Baker TR, Baldocchi D, Bekker R, Blanco CC, Blonder B, Bond WJ, Bradstock R, Bunker DE, Casanoves F, Cavender-Bares J, Chambers JQ, Chapin Iii FS, Chave J, Coomes D, Cornwell WK, Craine JM, Dobrin BH, Duarte L, Durka W, Elser J, Esser G, Estiarte M, Fagan WF, Fang J, Fernández-Méndez F, Fidelis A, Finegan B, Flores O, Ford H, Frank D, Freschet GT, Fyllas NM, Gallagher RV, Green WA, Gutierrez AG, Hickler T, Higgins SI, Hodgson JG, Jalili A, Jansen S, Joly CA, Kerkhoff AJ, Kirkup D, Kitajima K, Kleyer M, Klotz S, Knops JMH, Kramer K, Kühn I, Kurokawa H, Laughlin D, Lee TD, Leishman M, Lens F, Lenz T, Lewis SL, Lloyd J, Llusiá J, Louault F, Ma S, Mahecha MD, Manning P, Massad T, Medlyn BE, Messier J, Moles AT, Müller SC, Nadrowski K, Naeem S, Niinemets Ü, Nöllert S, Nüske A, Ogaya R, Oleksyn J, Onipchenko VG, Onoda Y, Ordoñez J, Overbeck G, Ozinga WA, Patiño S, Paula S, Pausas JG, Peñuelas J, Phillips OL, Pillar V, Poorter H, Poorter L, Poschlod P, Prinzing A, Proulx R, Rammig A, Reinsch S, Reu B, Sack L, Salgado-Negret B, Sardans J, Shiodera S, Shipley B, Siefert A, Sosinski E, Soussana JF, Swaine E, Swenson N, Thompson K, Thornton P, Waldram M, Weiher E, White M, White S, Wright SJ, Yguel B, Zaehle S, Zanne AE, Wirth C. 2011. TRY - a global database of plant traits. *Global Change Biology* 17:2905-2935.

Kissling WD, Dalby L, Fløjgaard C, Lenoir J, Sandel B, Sandom C, Trøjelsgaard K, Svenning J-C. 2014. Establishing macroecological trait datasets: digitalization, extrapolation, and validation of diet preferences in terrestrial mammals worldwide. *Ecology and Evolution* 4:2913-2930.

Kleyer M, Bekker RM, Knevel IC, Bakker JP, Thompson K, Sonnenschein M, Poschlod P, Van Groenendael JM, Klimes L, Klimesovâ J, Klotz S, Rusch GM, Hermy M, Adriaens D, Boedeltje G, Bossuyt B, Dannemann A, Endels P, Götzenberger L, Hodgson JG, Jackel A-K, Kühn I, Kunzmann D, Ozinga WA, Römermann C, Stadler M, Schlegelmilch J, Steendam HJ, Tackenberg O, Wilmann B, Cornelissen JHC, Erickson O, Garnier E, Peco B. 2008. The LEDA Traitbase: A database of life-history traits of Northwest European flora. *Journal of Ecology* 96:1266-1274.

Kranstauber B, Cameron A, Weinzerl R, Fountain T, Tilak S, Wikelski M, Kays R. 2011. The Movebank data model for animal tracking. *Environmental Modelling & Software* 26:834-835.

Mello MAR, Marquitti FMD, Guimaraes PR, Kalko EKV, Jordano P, de Aguiar MA. 2011a. The missing part of seed dispersal networks: Structure and robustness of bat-fruit interactions. *PloS One* 6:e17395.

Mello MAR, Marquitti FMD, Guimaraes PR, Kalko EKV, Jordano P, de Aguiar MA. 2011b. The modularity of seed dispersal: differences in structure and robustness between bat- and bird-fruit networks. *Oecologia* 167:131-140.

National Ecological Observatory Network. Battelle, Boulder, CO, USA. Available at http://data.neonscience.org. (data downloaded on).

Pigot AL, Bregman T, Sheard C, Daly B, Etienne RS, Tobias JA. 2016. Quantifying species contributions to ecosystem processes: a global assessment of functional trait and phylogenetic metrics across avian seed-dispersal networks. *Proceedings of the Royal Society B: Biological Sciences* 283:20161597.

Poelen J, Gosnell S, Slyusarev S. 2018. *rglobi: R Interface to Global Biotic Interactions*. R package version 0.2.17.

Razafindratsima OH, Yacoby Y, Park DS. 2018. MADA: Malagasy Animal trait Data Archive. *Ecology* 99:990-990.

Royal Botanic Gardens Kew. 2016. *Seed Information Database (SID)*. Available from: http://data.kew.org/sid/ (August 2016).

Salguero-Gómez R, Jones OR, Archer CR, Bein C, Buhr H, Farack C, Gottschalk F, Hartmann A, Henning A, Hoppe G, Römer G, Ruoff T, Sommer V, Wille J, Voigt J, Zeh S, Vieregg D, Buckley YM, Che-Castaldo J, Hodgson D, Scheuerlein A, Caswell H, Vaupel JW. 2016. COMADRE: a global data base of animal demography. *Journal of Animal Ecology* 85:371-384.

Salguero-Gómez R, Jones OR, Archer CR, Buckley YM, Che-Castaldo J, Caswell H, Hodgson D, Scheuerlein A, Conde DA, Brinks E, de Buhr H, Farack C, Gottschalk F, Hartmann A, Henning A, Hoppe G, Römer G, Runge J, Ruoff T, Wille J, Zeh S, Davison R, Vieregg D, Baudisch A, Altwegg R, Colchero F, Dong M, de Kroon H, Lebreton J-D, Metcalf CJE, Neel MM, Parker IM, Takada T, Valverde T, Vélez-Espino LA, Wardle GM, Franco M, Vaupel JW. 2015. The COMPADRE Plant Matrix Database: an open online repository for plant demography. *Journal of Ecology* 103:202-218.

Sinnott-Armstrong MA, Downie AE, Federman S, Valido A, Jordano P, Donoghue MJ. 2018. Global geographic patterns in the colours and sizes of animal-dispersed fruits. *Global Ecology and Biogeography* 27:1339-1351.

Tamme R, Götzenberger L, Zobel M, Bullock JM, Hooftman DAP, Kaasik A, Pärtel M. 2014. Predicting species' maximum dispersal distances from simple plant traits. *Ecology* 95:505-513.

Wikelski M, Kays R. *Movebank: archive, analysis and sharing of animal movement data.* . Movebank Data Repository, Max Planck Institute for Ornithology. Available at www.movebank.org. (data downloaded on September 2018).

Wilman H, Belmaker J, Simpson J, de la Rosa C, Rivadeneira MM, Jetz W. 2014. EltonTraits 1.0: Species-level foraging attributes of the world's birds and mammals. *Ecology* 95:2027-2027.
